# A Community Space With Diverse Activities Support Older Adults’ Social Participation and Sustain Social Connection

**DOI:** 10.1093/geroni/igaa057.1400

**Published:** 2020-12-16

**Authors:** Dongyool Kim, Taichi Masuda, Shuichiro Higuma, Yerim Yang, Sayaka Terazawa, Mai Takase, Ryogo Ogino, Jun Goto

**Affiliations:** The University of Tokyo, Tokyo, Japan

## Abstract

Active older adults in Japan participate in multiple social activities to be socially involved. However, physical limitation and decline in enthusiasm due to ageing decrease their participation. Diverse activities should be available at one place, close to older adult’s residence, to sustain social connections. A community space was launched at Toyoshikidai housing complex (Kashwia, Japan) in February 2018. The place offers about 25 activities per month. This research aimed to elucidate the relationship between activity type and motivation for participation, and study the effect of the community space on older adults’ social connection. A cross-sectional questionnaire survey was conducted targeting the attendees of community space (February 2020). Of attendees, 68% lived within 10-minutes walking distance to the community space (N=101). The activities were classified into craft, exercise, and music. The motivation for attending craft events were information exchange and relaxation, as was health maintenance for exercise events. Participating in group performance was the motivation to attend active music event, and casual gathering and network expansion was for passive music event. The frequency of social participation outside the community space was low in the group aged over 75 years. This group attended the activities at the community space more frequently than did the younger group. Differences in the number of social connections were not found. This result implies that older adults maintained their social connection by attending activities held at the community space. The diverse programs and close location of the community space might have contributed to the motivation of participation.

